# Deciphering Dormant Cells of Lung Adenocarcinoma: Prognostic Insights from O-glycosylation-Related Tumor Dormancy Genes Using Machine Learning

**DOI:** 10.3390/ijms25179502

**Published:** 2024-08-31

**Authors:** Chenfei Dong, Yang Liu, Suli Chong, Jiayue Zeng, Ziming Bian, Xiaoming Chen, Sairong Fan

**Affiliations:** 1 Key Laboratory of Laboratory Medicine, Ministry of Education, School of Laboratory Medicine and Life Sciences, Wenzhou Medical University, Wenzhou 325035, Chinaly228369@163.com (Y.L.); 15040365607@163.com (S.C.); 15397320562@163.com (J.Z.); bzm540147@163.com (Z.B.); 2 Institute of Glycobiological Engineering, School of Laboratory Medicine and Life Sciences, Wenzhou Medical University, Wenzhou 325035, China; 3 Wenzhou Key Laboratory of Cancer Pathogenesis and Translation, School of Laboratory Medicine and Life Sciences, Wenzhou Medical University, Wenzhou 325035, China

**Keywords:** lung adenocarcinoma, tumor dormancy, glycosylation, fibroblasts, IGF signaling, prognosis

## Abstract

Lung adenocarcinoma (LUAD) poses significant challenges due to its complex biological characteristics and high recurrence rate. The high recurrence rate of LUAD is closely associated with cellular dormancy, which enhances resistance to chemotherapy and evasion of immune cell destruction. Using single-cell RNA sequencing (scRNA-seq) data from LUAD patients, we categorized the cells into two subclusters: dormant and active cells. Utilizing high-density Weighted Gene Co-expression Network Analysis (hdWGCNA) and pseudo-time cell trajectory, aberrant expression of genes involved in protein O-glycosylation was detected in dormant cells, suggesting a crucial role for O-glycosylation in maintaining the dormant state. Intercellular communication analysis highlighted the interaction between fibroblasts and dormant cells, where the Insulin-like Growth Factor (IGF) signaling pathway regulated by O-glycosylation was crucial. By employing Gene Set Variation Analysis (GSVA) and machine learning, a risk score model was developed using hub genes, which showed high accuracy in determining LUAD prognosis. The model also demonstrated robust performance on the training dataset and excellent predictive capability, providing a reliable basis for predicting patient clinical outcomes. The group with a higher risk score exhibited a propensity for adverse outcomes in the tumor microenvironment (TME) and tumor mutational burden (TMB). Additionally, the 50% inhibitory concentration (IC50) values for chemotherapy exhibited significant variations among the different risk groups. In vitro experiments demonstrated that *EFNB2*, *PTTG1IP*, and *TNFRSF11A* were upregulated in dormant tumor cells, which also contributed greatly to the diagnosis of LUAD. In conclusion, this study highlighted the crucial role of O-glycosylation in the dormancy state of LUAD tumors and developed a predictive model for the prognosis of LUAD patients.

## 1. Introduction

Lung adenocarcinoma (LUAD) represents the most prevalent subtype of non-small-cell lung cancer (NSCLC), a significant global health concern that constitutes about 85% of all lung cancer cases [1]. LUAD is difficult to treat due to its high aggressiveness and metastasis. Despite significant progress in targeted therapy and immunotherapy, most patients still experience drug resistance and relapse following initial treatment, posing significant challenges to therapeutic strategies [2]. Patient recurrence can be attributed to treatment-surviving residual tumor cells (RTCs) [3]. These cells may exist in a reversible quiescent state, known as non-proliferative cellular dormancy [4]. Identifying the survival mechanisms of these critical cell pools is an attractive strategy for identifying additional potential therapeutic targets.

Tumor dormancy represents a critical stage in cancer progression, where tumor cells persist but clinical progression of the tumor is not apparent [5]. Tumor dormancy, which has been extensively studied across various cancer types, significantly affects disease progression and patient prognosis [6,7]. Understanding how cancer cells maintain dormancy and what triggers their reactivation is crucial for improving patient outcomes and developing more effective treatment strategies. In recent years, substantial advancements have been achieved in uncovering the mechanisms underlying tumor dormancy [8,9]. For instance, DDR1, a collagen receptor previously proven to be essential for tumor cell dormancy [10], regulates the cell cycle arrest of RTCs, preventing their expansion, despite studies showing that regardless of the stimulus or location, the gene expression profiles of dormant RTCs are remarkably similar [11,12], and the expression characteristics and underlying mechanism of dormant tumor cells remain limited.

Glycosylation, the process by which sugars are chemically attached to proteins or lipids, influences a variety of cellular functions. The complex “glycan coding” on the surface of tumor cells significantly influences cancer development by interfering with immune system surveillance [13], regulating the cell cycle [14,15], and reshaping the tumor microenvironment (TME) [16,17], which potentially plays critical roles in maintaining tumor cell dormancy. Sreekumar et al. confirmed that B3GALT6 mediated the linkage of heparan sulfate (HS) to proteoglycans, which play a crucial role in the tumor microenvironment-induced dormancy model [18]. Recent research indicates that post-modification is crucial in sustaining the dormancy of RTCs [19,20]; however, how glycosylation regulates cellular dormancy remains largely unexplored.

Here, we used bioinformatics analysis to explore the prognostic value of O-glycosylation-related tumor dormancy genes (ORDGs) in LUAD. Based on the selected ORDGs, we developed a novel prognostic risk model that effectively predicts the prognosis and immunotherapy response of LUAD patients. Our analysis explored the differences in genomic variations, drug sensitivity, and the tumor microenvironment between the two risk groups, aiming to provide new insights and targeted therapeutic approaches for LUAD.

## 2. Results

### 2.1. Investigating the Prognostic Impact of Tumor Dormancy-Associated Genes across Cancer Types

**Figure 1:**
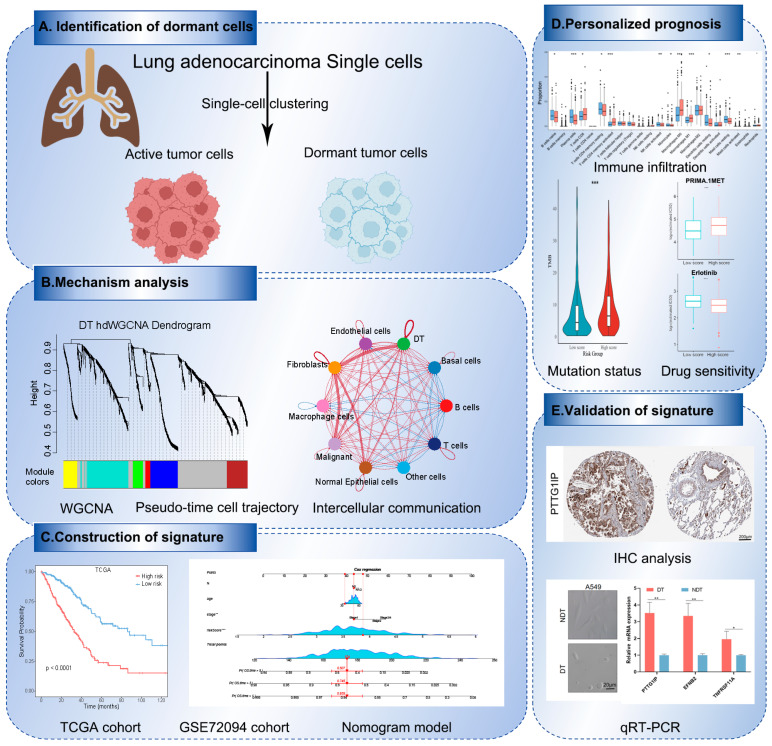
The workflow demonstrating the research idea of this paper. * *p* < 0.05, ** *p* < 0.01, *** *p* < 0.001.

Figure 1 presents a schematic that details the basic analytical methodology utilized in this study. We carried out a comprehensive and integrative analysis of 46 genes associated with tumor dormancy to determine their prognostic value for cancer patients (Table S1). Hazard ratio analysis was used to assess the prognostic performance of these genes in various cancers (Figure S1A). Forty-six dormancy-associated genes were used as gene signatures to analyze survival outcomes in patients with different cancers. The results showed that tumor dormancy generally presented a favorable prognostic indicator across multiple cancer types, including LUAD (Figure S1B).

### 2.2. Identification of Dormant and Active Cell Clusters in LUAD

**Figure 2:**
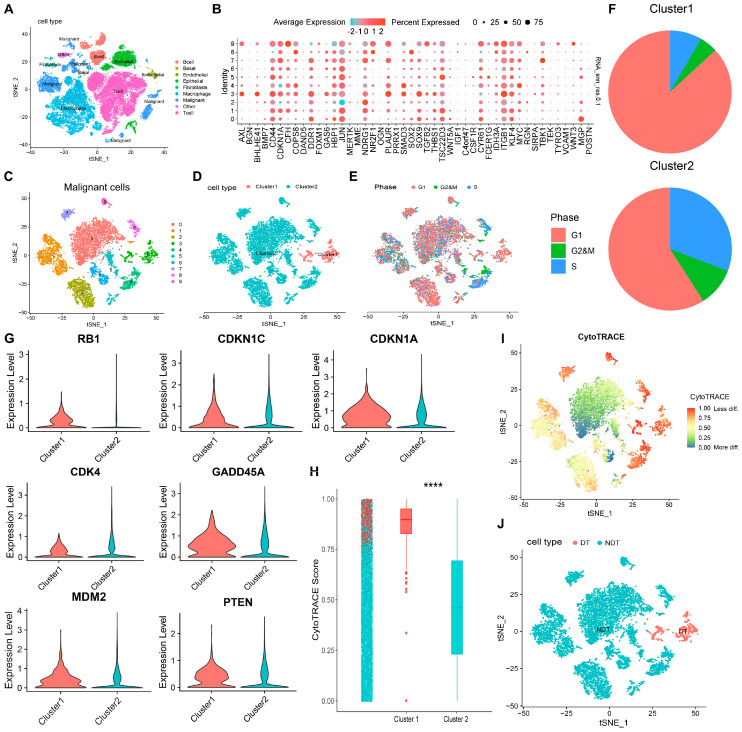
Identification of the DT group in LUAD scRNA-seq data. (**A**) The t-SNE plot of the distribution of the nine cell types. (**B**) Bubble plots of the expression of dormancy-related genes in cells in different clusters. (**C**) The t-SNE plot colored according to 10 cell subpopulations. (**D**) The t-SNE plot colored according to 2 cell subpopulations. (**E**,**F**) Assessment of the cell cycle in the scRNA-seq data. (**G**) Violin map of cell cycle genes in the cluster1 and cluster2 groups. (**H**,**I**) Assessment of stemness in the scRNA-seq data (**** *p* < 0.0001). (**J**) The t-SNE plot of the distribution of the 2 cell types.

To explore the specific characteristics of dormant LUAD tumor cells, we used single-cell RNA sequencing (scRNA-seq) analysis. The scRNA-seq data from LUAD patients encompassed 57,166 cells, featuring key cell types such as B cells, basal cells, epithelial cells, endothelial cells, fibroblasts, macrophages, malignant cells, and T cells (Figure 2A) [21]. Based on the 46 dormancy-associated genes, the malignant cells of LUAD were categorized into two distinct clusters by single-cell clustering (Figure 2B–D and Figure S2A). Compared to those in Cluster 2, cells in Cluster 1 exhibited significant cell cycle arrest (Figure 2E–G) and enhanced tumorigenic stem cell characteristics (Figure 2H,I). Based on the above results, cluster 1 was defined as the DT group, and cluster 2 was defined as the NDT group (Figure 2J). Dormant tumor cells with immune escape properties to evade immune destruction are thought to contribute to cancer progression [20]. Significant variations were observed in the expression levels of immune checkpoint genes between the DT group and the Non-Dormant (NDT) group (Figure S2B). Specifically, many Human Leukocyte Antigen (HLA) class molecules, which aid tumor cells in evading T-cell-mediated immune responses [22], were expressed at lower levels in the DT group. The expression of immune checkpoint molecules, such as CD40 and CD276, was higher in the DT group compared to the NDT group (Figure S2C).

### 2.3. Analysis of the Correlation between O-glycosylation and Tumor Dormancy

**Figure 3:**
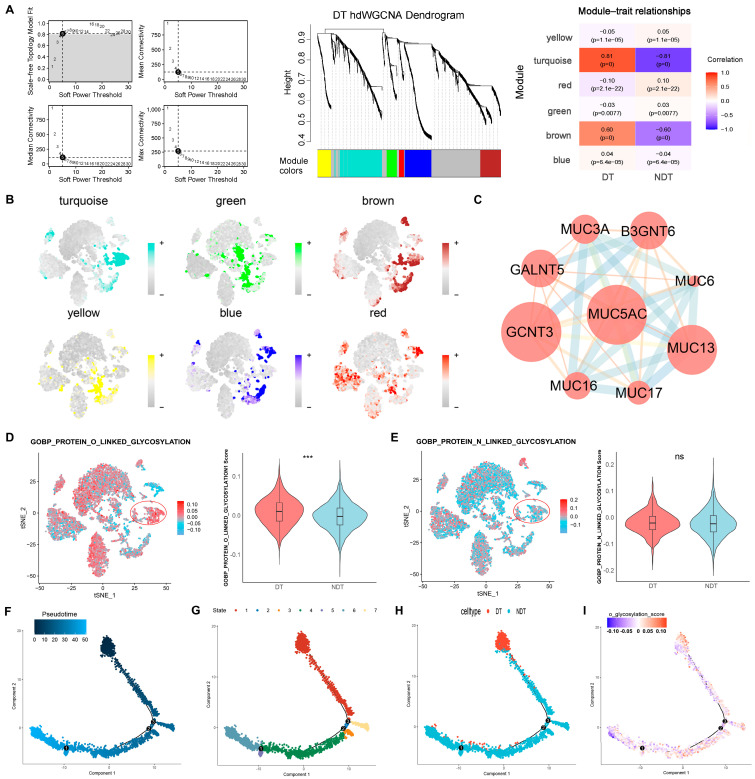

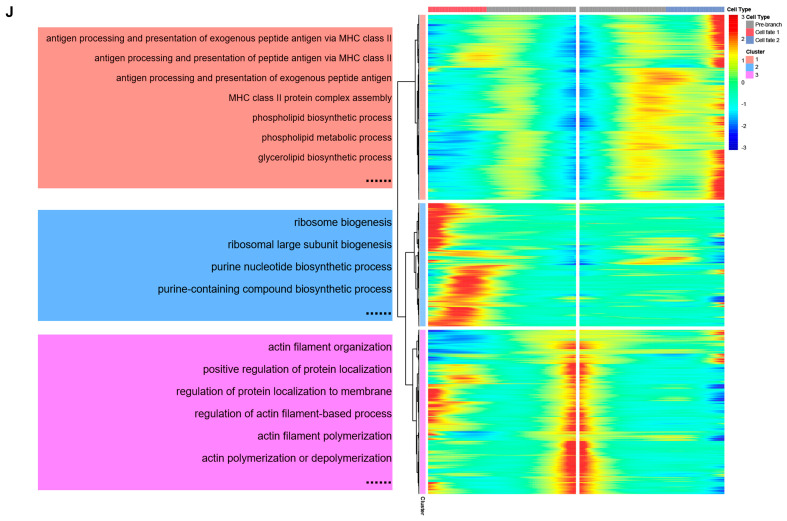
Relationship between O-glycosylation and tumor dormancy according to the LUAD scRNA-seq data. (**A**) The gene sets most strongly correlated with the occurrence of DT were identified via hdWGCNA. (**B**) The t-SNE plots of the expression distribution for the different colored gene modules. (**C**) The PPI plots of protein O-linked glycosylation in the DT-associated gene sets. (**D**,**E**) Comparison of O-glycosylation (**D**) and N-glycosylation (*** *p* < 0.001) (**E**) between the DT and NDT groups. (**F**) Pseudo-time was represented with a color gradient that transitions from dark blue to light blue. (**G**) Pseudo-time trajectory was segmented into seven distinct states using Monocle 2. (**H**) Pseudo-time trajectory divided into DT and NDT groups. (**I**) Pseudo-time trajectory analysis based on AddModule scores shows that O-glycosylation levels change along the pseudo-time trajectory. (**J**) Heatmap showing differentially expressed genes (DEGs) across various branches (cell fate), with enriched Gene Ontology (GO) pathways annotated to the left of each gene cluster.

To gain insight into the intrinsic regulatory mechanisms of tumor dormancy, we conducted high-density Weighted Gene Co-expression Network Analysis (hdWGCNA) on the malignant cluster to identify the gene modules closely related to dormancy (Figure 3A,B). Subsequent enrichment analysis of the genes revealed that a range of protein O-glycosylation genes exhibited high expression levels in the DT group (Figure 3C). The levels of O-glycosylation were significantly elevated in the DT group, while the N-glycosylation levels were not significantly different (Figure 3D,E).

Furthermore, we developed a cell trajectory analysis shown in Figure 3F to explore the dynamics of O-glycosylation and gene expression within the malignant cell cluster. The analysis of transition states within the trajectory revealed that the DT group was predominantly occupied in state 1 (Figure 3G,H). As shown in Figure 3I, significant fluctuations in the O-glycosylation score were observed throughout the trajectory, where the O-glycosylation levels in cell fate 1 (state 1) remained more constant compared to those in the pre-branch. To further understand the molecular mechanisms behind these transitions, we investigated genes influencing the branching into dormant tumor cell fates. In the pre-branch phase, genes were mainly linked to the biological processes involving “actin filament” and “localization of regulatory proteins” (Gene Ontology terms). Meanwhile, pathways such as “ribosome” and “purine nucleotide synthesis” were significantly represented in cell fate 1. In contrast, cell fate 2 was characterized by genes related to “class II MHC protein complex” and “phospholipid biosynthesis” (Figure 3J and Table S2).

### 2.4. Intercellular Communication between Malignant Cells with Fibroblasts Involved in Tumor Dormancy

**Figure 4:**
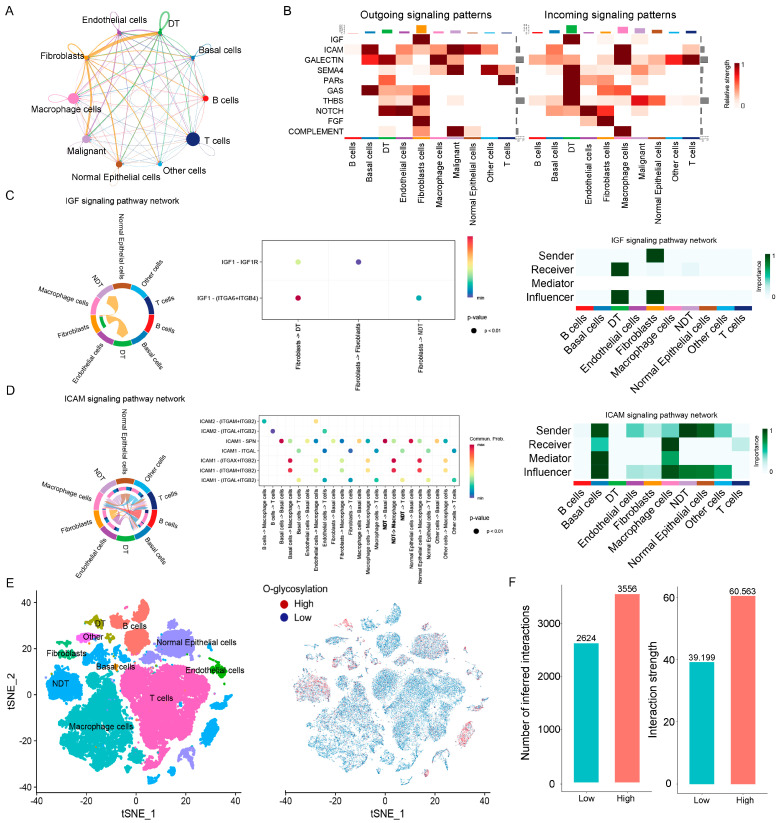

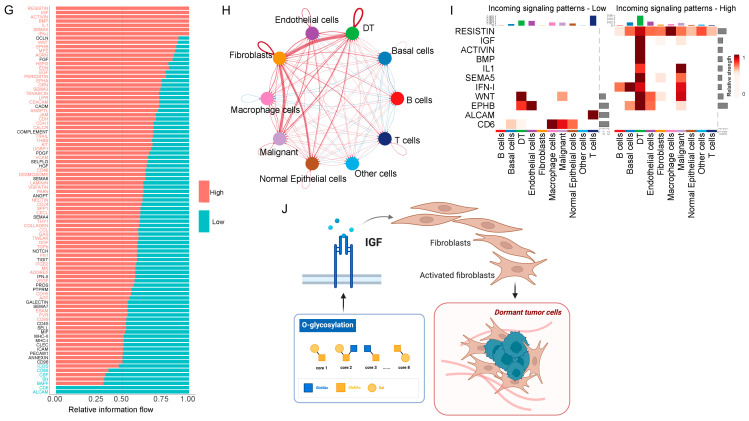
Cell–cell communication analysis of DT cells in LUAD. (**A**) Number of interactions among cell subtypes. (**B**) Heatmap of signaling pathways. (**C**,**D**) Comparison of the ligand–receptor pairs related to IGF (**C**) and ICAM (**D**) signaling pathways in LUAD cells. (**E**) The t-SNE plots of 57,166 cells classified by O-glycosylation genes. High, high O-glycosylation subtype; Low, low O-glycosylation subtype. (**F**) Number and intensity of interactions between high and low O-glycosylation cell types. (**G**) Overview of the comparison of ligand–receptor interactions between high and low O-glycosylation groups. Red represents the high O-glycosylation group having more interactions than the low group, otherwise colored blue. (**H**) Bar plot of the relative proportion of interaction strengths for each signal between the high and low O-glycosylation groups. (**I**) Heatmap of the incoming signal across two groups. (**J**) Schematic representation of O-glycosylation linking fibroblast action with DT cells by regulating the IGF signaling pathway.

To further investigate the functional dynamics of dormant tumor cells within the TME, we utilized “CellChat” to analyze the interactions between DT cells and other cell types (Figure 4A). We observed that the interactions between DT cells and cancer-associated fibroblasts (CAFs) were markedly more significant compared to those between CAFs and NDT cells, highlighting the involvement of CAFs in the dormancy mechanisms of LUAD cells. Figure 4B illustrates the patterns of signal reception and transmission across different cell types. We noted significant differences in the incoming signaling pattern of the Insulin-like Growth Factor (IGF) between DT and NDT cells, with CAFs primarily serving as the major signal sender source (Figure 4C). Additionally, the ICAM signaling pathway, associated with cell adhesion functions, did not impact DT cells, whereas NDT cells were a significant sender source of the ICAM signal (Figure 4D), which might relate to the stealth characteristics of DT cells.

Due to the significantly elevated levels of O-glycosylation in DT cells, we further explored the mechanisms of O-glycosylation’s impact on DT cells. Utilizing the “AddModuleScore” function, each cell was assessed for O-glycosylation levels and categorized into high or low groups (Figure 4E). The high group exhibited increased total numbers and intensities of cell interactions (Figure 4F,G), with notable differences in signaling pathways such as RESISTIN, IGF, BMP, ALCAM, and CD6 between the two groups (Figure 4H). Notably, the IGF pathway exhibited information flow exclusively within the high O-glycosylation pattern, and cellular interactions were more pronounced in this group. DT cells in the high O-glycosylation group were the main receivers of the IGF pathway (Figure 4I). Taken together, CAFs may mediate the dormancy mechanisms of DT cells through the IGF signaling pathway, while O-glycosylation enhances the transmission of the IGF signal (Figure 4J).

### 2.5. Construction of a Prognostic Model Based on ORDGs

**Figure 5:**
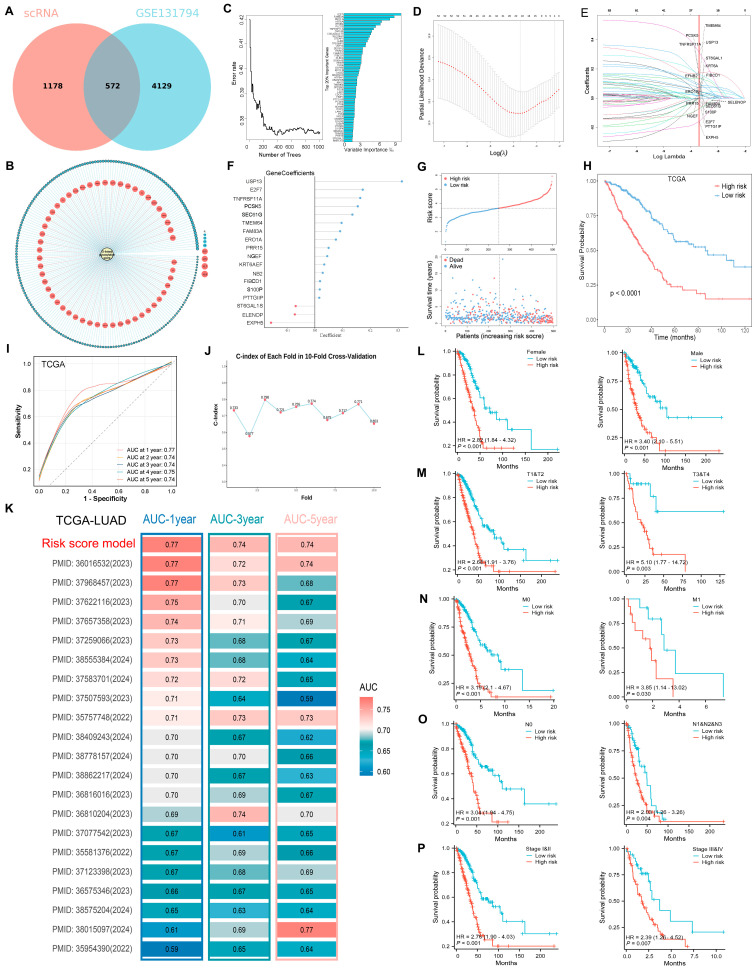
Construction of the ORDG-related risk score model in the TCGA-LUAD cohort. (**A**) Venn diagram showing an overlap between 1750 single-cell DEGs and 4701 GSE131594 DEGs, resulting in the identification of 572 HUB genes. (**B**) The correlation between the O-glycosylation score and HUB gene expression. (**C**) Random forest analysis of the genes related to O-glycosylation. (**D**,**E**) Cvift (**D**) and lambda (**E**) curves of LASSO regression applied with minimum criteria. (**F**) Coefficients of the genes in the prognostic model. (**G**) Survival status of the high- and low-risk groups. (**H**) K–M curves of the prognostic model in the TCGA cohort. (**I**) ROC curves of the LUAD patients. (**J**) The consistency index (C-index) indicated the predictive effect. (**K**) Heatmaps showing the AUC values of the ROC curve at 1, 3, and 5 years for different prognostic models based on the TCGA-LUAD dataset. (**L**–**P**) K–M curves stratified the differences in OS between high- and low-risk groups by gender (**L**), T (**M**), M (**N**), N (**O**), and pathological stage (**P**).

We identified 1750 DEGs with significant differences (|log (2) fold change| > 1; adjust *p* < 0.05) between the DT and NDT clusters in the single-cell dataset. These DEGs were then intersected with 4701 DEGs identified between the DT and NDT groups in the GSE131594 dataset [23] (|log (2) fold change| > 1; adjust *p* < 0.05), resulting in a set of 572 overlapping genes (Figure 5A). Additionally, the DT group exhibited greater drug resistance than did the NDT group in GSE131594 (Figure S3). Given the regulatory role of O-glycosylation in tumor dormancy, we performed an O-glycosylation Gene Set Variation Analysis (GSVA) on LUAD samples from the Cancer Genome Atlas (TCGA), filtering out 266 genes with a correlation greater than 0.1 and *p* value < 0.05 to the O-glycosylation score (Figure 5B and Table S3). Furthermore, we utilized the random forest SRC algorithm for further analysis of these 266 genes, selecting the top 20% based on importance, which resulted in the identification of 53 O-glycosylation-related tumor dormancy genes (Figure 5C). Finally, using TCGA-LUAD as the training set, we applied Subsequent Least Absolute Shrinkage and Selection Operator (LASSO) regression analysis (Figure 5D,E) to select 18 genes from these 53 genes to construct a prognostic model, along with their corresponding coefficients (Figure 5F). Subsequently, the risk score for each LUAD patient within the TCGA cohort was determined using the previously mentioned formula, and subsequently, patients were segregated into high-risk and low-risk groups according to the median risk score. Analysis of survival status revealed that the prognosis for the high-risk group was less favorable compared to the low-risk group (Figure 5G). K–M curves showed that overall survival (OS) was significantly lower for patients in the high-risk group (Figure 5H). In the TCGA cohort, the receiver operator characteristic (ROC) of our constructed risk model demonstrated the area under the curve (AUC) values of 0.77 at 1 year, 0.74 at 2 years, 0.74 at 3 years, 0.75 at 4 years, and 0.74 at 5 years (Figure 5I). A stable concordance index (C-index) confirmed that the model had good prognostic ability (Figure 5J). Moreover, compared to other prognostic models established in the TCGA-LUAD cohort, the ORDG prognostic model showed an advantage in predicting overall survival at 1, 3, and 5 years (Figure 5K). Stratified analysis showed that LUAD patients with a high-risk score tended to have poorer long-term survival across different subgroups, including females and males, T1&T2 and T3&T4, M0 and M1, N0 and N1&N2&N3, and stages I&II and III&IV (Figure 5L–P).

### 2.6. Validation of the Model and Establishment of the Prognostic Nomogram

**Figure 6:**
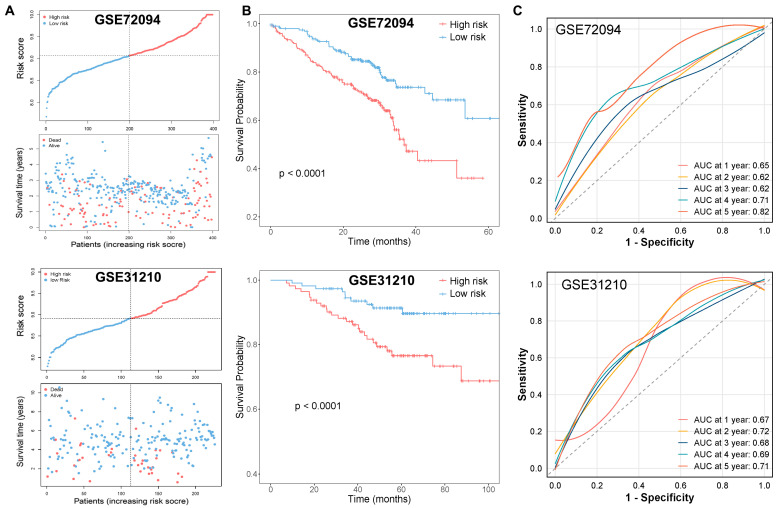

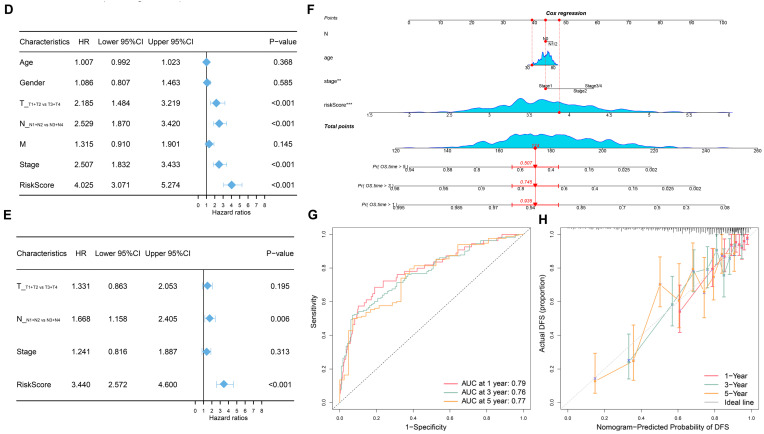
Validation of the risk model and construction of the nomogram for LUAD patients. (**A**) Risk score and survival status of LUAD patients in GSE72094 and GSE31210. (**B**) K–M curves of the prognostic model in the GEO validation cohort (GSE72094 and GSE31210). (**C**) ROC curves of the validation cohort (GSE72094 and GSE31210). (**D**,**E**) Univariate (**D**) and multivariate (**E**) Cox analyses of clinical characteristics and prognostic models. (**F**) Nomogram for predicting the 1-, 3-, and 5-year OS rates (** *p* < 0.01, *** *p* < 0.001). (**G**) ROC curves of the nomogram. (**H**) Calibration curve for assessing the accuracy of the nomogram in predicting the OS rates.

We validated the model using the GSE72094 and GSE31210 dataset, where, consistent with the TCGA training set, patients with high-risk scores exhibited poor prognosis (Figure 6A,B): for the GSE72094 dataset, the AUC values of 0.65 at 1 year, 0.62 at 2 years, 0.62 at 3 years, 0.71 at 4 years, and 0.82 at 5 years (Figure 6C), confirming the model’s reliability in predicting LUAD prognosis; in the GSE31210 dataset, the AUC values of 0.67 at 1 year, 0.72 at 2 years, 0.68 at 3 years, 0.69 at 4 years, and 0.71 at 5 years (Figure 6C), further validating its predictive accuracy.

Additionally, heatmaps were generated to display the profiles of the 18 genes in both groups in the TCGA-LUAD dataset (Figure S4A), and a Sankey diagram was employed to illustrate the relationships between risk scores and pathological characteristics (Figure S4B). The results showed that LUAD patients with high-risk scores were associated with more advanced T, N, M, and pathological stages (Figure S4C), suggesting that this model can predict the prognosis of LUAD patients. Furthermore, we utilized univariate Cox analysis, which indicated that the risk score, T stage (tumor size), N stage (node involvement), and pathological stage were associated with the long-term survival of LUAD patients (Figure 6D). Multivariate analysis further confirmed that the risk score and N concentration were independent prognostic factors, significantly impacting long-term survival (Figure 6E). A nomogram was then constructed based on each patient’s N stage, pathological stage, age, and risk score to visually predict long-term survival (Figure 6F). The AUCs of the nomograms for predicting the 1-, 3-, and 5-year survival of TCGA-LUAD patients were 0.79, 0.76, and 0.77, respectively (Figure 6G). Calibration curves confirmed the nomogram model’s precise predictive accuracy at these time points (Figure 6H).

### 2.7. Functional Enrichment Analyses

**Figure 7:**
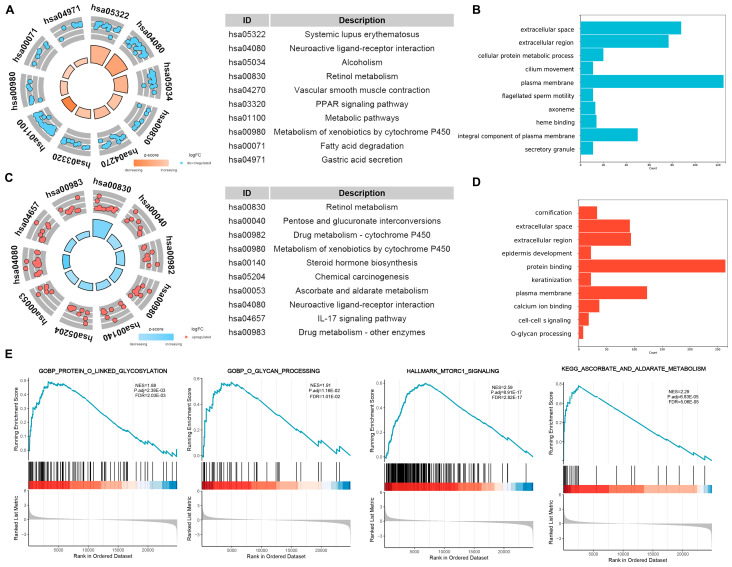
Enrichment analyses of the ORDG-related prognostic model. (**A**,**B**) KEGG (**A**) and GO (**B**) enrichment analyses of the highly expressed genes in the low-risk group (showing the top 10). (**C**,**D**) KEGG (**C**) and GO (**D**) enrichment analyses of the highly expressed genes in the low-risk group (showing the top 10). (**E**) GSEA across risk groups.

In the high-risk group, 835 genes were upregulated (|log (2) fold change| > 1; adjust *p* < 0.05), whereas in the low-risk group, 1189 genes were upregulated under the same criteria. We utilized the KOBAs bioinformatics resource [24] to perform functional enrichment analysis to investigate the potential biological functions of these highly expressed genes in both risk groups. In the low-risk group, metabolic pathways, metabolism of xenobiotics by cytochrome P450, and the PPAR signaling pathway were enriched (Figure 7A). Functions analysis showed that cellular protein metabolic processes and localization to the extracellular space were primarily involved (Figure 7B). Conversely, in the high-risk group, retinol metabolism and drug metabolism cytochrome P450 were the dominant pathways (Figure 7C), emphasizing biological processes such as protein binding and cell–cell signaling (Figure 7D). We conducted GSEA on DEGs between risk score groups using the “ClusterProfiler” package, and the GSEA results indicated that the risk scores were significantly associated with MTORC1 signaling and protein O-linked glycosylation (Figure 7E).

### 2.8. Analysis of the Immunological Profile

**Figure 8:**
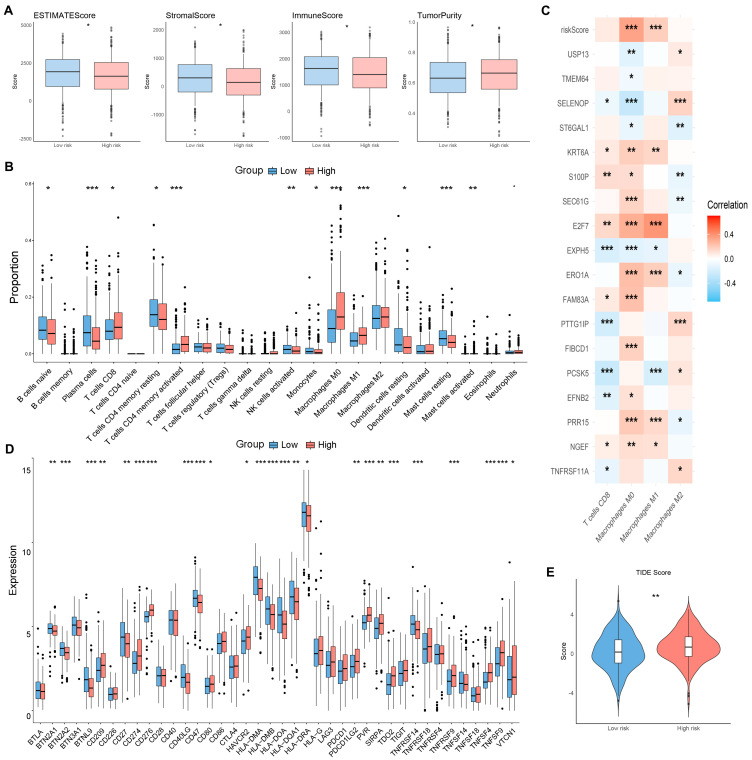
Immune profile analyses of the ORDG-related prognostic models. (**A**) Comparison of the TME among risk groups. (**B**) Box plots of the degree of immune cell infiltration. (**C**) The correlation of the risk score with M0 macrophages, CD8 T cells, M2 macrophages, and M1 macrophages. (**D**) Comparison of 40 immune checkpoint genes. (**E**) Comparison of TIDE. * *p* < 0.05, ** *p* < 0.01, *** *p* < 0.001.

Given that the TME-associated signaling pathway was enriched, we employed the ESTIMATE algorithm to analyze the stromal scores, immune scores, and ESTIMATE scores of LUAD patients. The results showed that the high-risk group exhibited significantly lower stromal scores, immune scores, and ESTIMATE scores, indicating a greater level of tumor purity in this group (Figure 8A). To analyze immune infiltration across risk groups, we employed the CIBERSORT algorithm [25], which estimates the relative proportions of tumor-infiltrating immune cells (TIICs) by estimating the RNA transcriptome. In the high-risk group, there was a notable increase in the percentage of CD8 T cells, M0 macrophages, and M1 macrophages (Figure 8B). Conversely, the levels of resting memory CD4 T cells, naive B cells, and plasma cells were higher in the low-risk group than in the high-risk group (Figure 8B). The heatmap indicated that characteristic genes were associated with CD8 T cells, and most of the genes were positively correlated with the expression of M0 macrophages (Figure 8C).

Currently, immune checkpoint inhibitors have been extensively studied and applied in tumor immunotherapy [26,27]. In this study, we found that the expression of 25 immune checkpoint genes significantly changed across the risk groups (Figure 8D). The expression levels of PD-L1 and SIRPα were elevated in the high-risk group (Figure 8D), which is currently the focus of research on therapies for advanced LUAD [28,29]. Based on these findings, we used the Tumor Immune Dysfunction and Exclusion (TIDE) tool (http://tide.dfci.harvard.edu/, accessed on 1 June 2024) to analyze TCGA-LUAD patients. The results indicated that TIDE scores were increased in the high-risk group (Figure 8E), suggesting that patients in the high-risk group may respond poorly to immunotherapy. The results could be helpful for guiding personalized immunotherapy strategies to enhance the efficacy of treatment in patients.

### 2.9. Analysis of Mutation Status and Drug Sensitivity

**Figure 9:**
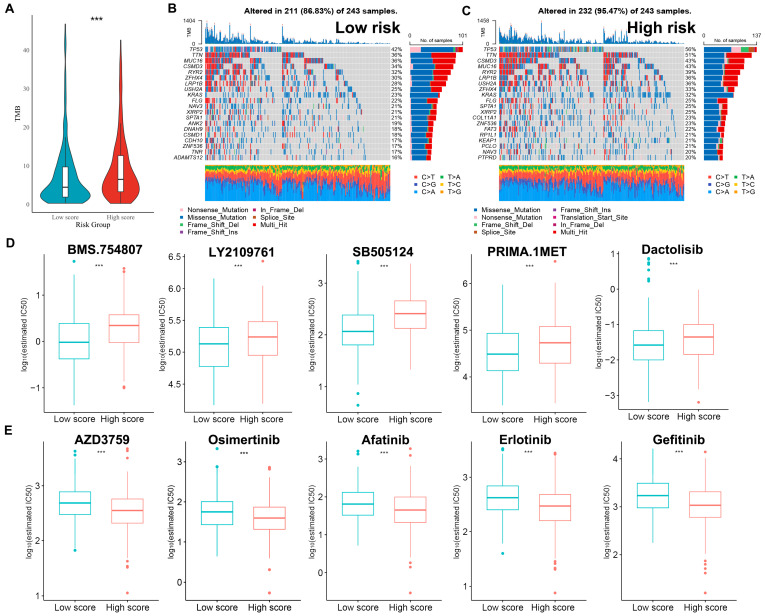
Cancer-related gene mutations and drug sensitivity in the risk model. (**A**) Comparison of TMB between the high-risk and low-risk groups. (**B**,**C**) Waterfall plot of the top 20 genes with mutations in the TCGA-LUAD low- (**B**) and high-risk groups (**C**). (**D**,**E**) Drugs that are more sensitive to low- (**D**) and high-risk (**E**) groups (*** *p* < 0.001).

The accumulation of gene mutations can lead to cancer and is closely related to cancer progression [30]. Genomic sequencing provides a detailed view of the mutation processes and genes driving cancer, enhancing our understanding of somatic mutations in cancer. Increasing evidence supports a role for tumor mutation burden (TMB) as a potential predictive biomarker in multiple applications [31]. The TMB was significantly greater in the high-risk group compared to the low-risk group (Figure 9A). By analyzing the top 20 most frequently mutated genes common across both groups, we found that mutations in *TP53*, *TTN*, *MUC16*, *CSMD3*, and *RYR2* were increased in the high-risk group (Figure 9B,C).

In actual clinical practice, chemotherapy is commonly combined with surgery, radiotherapy, and targeted therapy to optimize therapeutic outcomes [32,33]. As chemotherapy remains a vital treatment for LUAD, an analysis was performed to assess if the risk score could effectively predict the chemotherapy responses of patients. By assessing and comparing the drug sensitivity, Dactolisib might be suitable for patients with lower risk scores (Figure 9D), whereas EGFR inhibitors such as AZD3759, Osimertinib, and Gefitinib may be more appropriate for patients with higher risk scores (Figure 9E).

### 2.10. Validation of the Prognostic Model Genes

**Figure 10:**
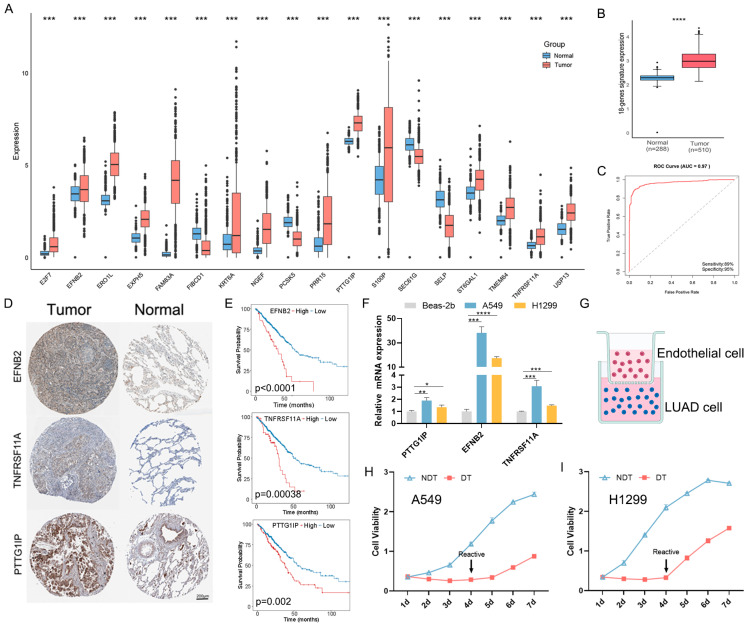

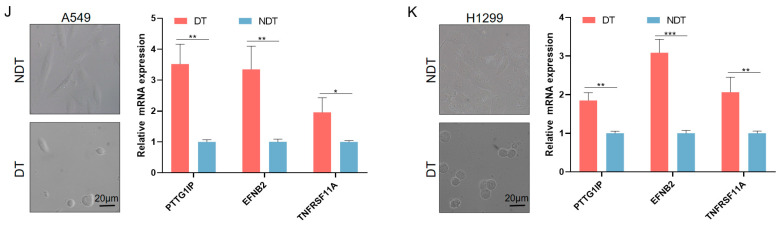
Validation of the expression levels of prognostic genes. (**A**) Expression levels of model prognostic genes in the TCGA + GTEx database. (**B**) Comparison of the level of the 18-gene signature. (**C**) ROC curve of 18-gene signature mRNA expression in LUAD (AUC, 0.97; sensitivity, 89%; specificity, 95%). (**D**) Comparison of the gene by immunohistochemistry between LUSC and normal samples. (**E**) The K–M curves for the group with high gene expression combined with the group with low expression. (**F**) qRT-PCR analysis of gene mRNA expression in LUAD cell lines (A549 and H1299) and Beas-2b cell lines. (**G**) Schematic diagram illustrating the construction of dormant cells from LUAD cells. (**H**,**I**) CCK8 analysis of DT and NDT groups in A549 (**H**) and H1299 (**I**) cell lines. (**J**,**K**) qRT-PCR analysis of gene mRNA expression of DT and NDT groups in A549 (**J**) and H1299 (**K**) cell lines. * *p* < 0.05, ** *p* < 0.01, *** *p* < 0.001, **** *p* < 0.0001.

To verify the reliability of the predicted model, we analyzed the mRNA levels of 18 genes in normal lung tissue specimens and LUAD specimens using data from TCGA and GTEx. The results revealed variations in the expression of these 18 genes when comparing normal and tumor specimens (Figure 10A), and the expression level of gene signature was significantly elevated in LUAD patients (Figure 10B). ROC curve analysis demonstrated an AUC of 0.97 (Figure 10C), indicating that these genes could facilitate the diagnosis of LUAD. The Immunohistochemistry (IHC) results from the Human Protein Atlas (HPA) showed that *EFNB2*, *PTTG1IP*, and *TNFRSF11A* were highly expressed in LUAD (Figure 10D), and the K–M survival analysis showed their prognostic value (Figure 10E). To validate differential gene expression, qRT-PCR compared mRNA levels in A549 and H1299 cells versus normal Beas-2b cells, showing increased transcripts in A549 and H1299 (Figure 10F). Furthermore, we established the in vitro dormancy pattern of LUAD cells (Figure 10G), and the results of cell viability analysis indicated that DT cell proliferation was significantly arrested, whereas proliferation was still restored after reactivation (Figure 10H,I), indicating the successful establishment of the dormant cell pattern. Notably, *EFNB2*, *PTTG1IP*, and *TNFRSF11A* were increased in DT cells compared to NDT cells (Figure 10J,K).

## 3. Discussion

LUAD ranks among the most lethal malignancies globally and is known for its high incidence and mortality rates [34,35]. Due to its severe impact, understanding the mechanisms underlying disease progression is crucial. Tumor dormancy represents a crucial phase in cancer progression and provides a vital window for therapeutic intervention [36]. The period of clinical dormancy raises numerous questions for patient management, as dormant tumor cells may have disseminated to other organs, such as the brain or liver, early in the disease and can cause recurrence years after primary tumor treatment [37,38]. Recent studies suggest that the intrinsic mechanisms of tumor cells and their interactions with the microenvironment jointly regulate entry and exit from dormancy [39]. Understanding the profiles of dormant cells may provide an effective approach to identifying dormant RTCs, which could be targeted and eliminated through interventions. Therefore, uncovering how tumor cells maintain dormancy and the mechanisms underlying waking has become a fundamental issue in oncology research [40].

In tumor diagnosis and prognosis, genetic alterations often precede evident histopathological changes, necessitating the development of genetics-based biomarkers [41,42]. There is an increasing demand for genes that can accurately predict the prognosis of LUAD patients. Here, using scRNA-seq and machine learning, we identified reliable tumor dormancy signatures as robust prognostic tools for LUAD. Detailed analysis of LUAD tumor heterogeneity classified specific clusters with arrested proliferation and elevated tumor stemness levels as dormant cells. These dormant cell clusters exhibited immune evasion traits and resistance to common chemotherapeutic agents. The results showed increased expression of molecules such as SIRPA and PVR in dormant cells, which affects immune cell activity through interactions with their receptors [43]. For example, SIRPA interacts with CD47 to provide a “do not eat me” signal [44], helping dormant tumor cells evade macrophage-mediated phagocytosis.

In this study, we conducted cell–cell communication analysis on specific cell types, enhancing our understanding of the biological context and interaction networks in tumor dormancy. Cancer-associated fibroblasts (CAFs), prevalent in the tumor microenvironment, have a proven role in regulating tumor dormancy [45]. Our findings indicate that dormant tumor cells interact with fibroblasts via the IGF pathways. IGF appears to mediate these interactions, influencing the complex dynamics among DT cells and other cell types. IGF regulates growth, differentiation, and survival by interacting with its receptors (IGF1R and IGF2R). Shimizu et al. have demonstrated that IGF2 induces autophagic dormancy, helping osteosarcoma cells withstand chemotherapy [46], while autocrine IGF1 signaling, in the absence of oncogenic drivers, mediates the dormancy of pancreatic tumor cells [47].

Recent studies have highlighted the essential role of protein glycosylation in maintaining tumor dormancy, offering new insights into targeting this pathway to eliminate RTCs and reduce tumor recurrence [48]. It has been reported that specific α2,6-sialylation catalyzed by ST6GAL1 in glycoproteins is closely connected with the chemotaxis of quiescent tumor cells towards metastatic seeding [49]. Our analysis revealed that the dormant cell clusters in LUAD exhibited abnormal glycosylation, particularly O-glycosylation. Moreover, we found that O-glycosylation is widely involved in intercellular communication in LUAD, where the high O-glycosylation status was significantly associated with the regulation of the IGF pathway. This seems to indicate the complex regulatory role of O-glycosylation on DT cells in LUAD, potentially enabling dormant tumor cells to evade immune detection and survive chemotherapy. Building on the established connection between O-glycosylation and the regulation of the IGF pathway in dormant LUAD cells, potential therapeutic strategies should focus on disrupting this glycosylation to prevent tumor recurrence. Targeting the enzymes involved in O-glycosylation, such as specific glycosyltransferases, could inhibit the abnormal glycosylation patterns observed in these cells. This strategy might sensitize dormant cells to immune surveillance and chemotherapy by exposing them to the host immune system and reducing their metabolic adaptability. Furthermore, combining O-glycosylation inhibitors with current chemotherapeutic regimens has shown significant potential in tumor therapy [50,51]. This dual approach, targeting both active and dormant tumor cells, could enhance patient outcomes by decreasing the likelihood of metastatic recurrence. However, additional preclinical studies and clinical trials are essential to confirm the efficacy and safety of this targeted therapy in the context of LUAD.

By applying GSVA, random forest SRC, and LASSO regression analyses, a prognostic model was constructed. This model, integrating weights for particular genes, was established as an independent prognostic factor for LUAD. Additionally, we developed a nomogram to visually predict OS at 1, 3, and 5 years for LUAD patients. To validate our findings, we utilized an external dataset to verify and predict prognostic outcomes, thereby enhancing the reliability of our results. This model successfully categorized LUAD patients into distinct prognostic groups, where the high-risk category demonstrated notably worse survival outcomes. Additionally, we analyzed genomic variations, differences in drug sensitivity, and differences in immune profiles between the high-risk and low-risk groups, offering comprehensive insights into the clinical relevance.

The tumor microenvironment is crucial in various stages of tumor development, metastasis, and immune evasion [52]. In LUAD patients, there were significant differences in the TME between different risk groups. The ESTIMATE scores were significantly greater in the low-risk group than in the high-risk group, indicating lower tumor purity. Variations in the activation of immune cell populations were noted, indicating that the risk score is associated with prognosis and reflects diverse immune responses within the TME. Our study revealed strong associations between the *E2F7*, *KRT6A*, *NGEF*, and *PRR15* genes and certain immune cells, offering perspectives on their involvement in modulating the immune response. Notably, based on our prediction, patients in the low-risk group identified by ORDGs might benefit more from immunotherapy.

To further explore its biological significance, we conducted pathway enrichment analysis. The patients with high-risk scores showed significant enrichment in drug metabolism-related pathways, including cytochrome P450 and various drug-metabolizing enzymes. The mutation frequencies of *TP53*, *MUC16*, and *TTN*, which have been linked to adverse outcomes in previous studies [53,54], were higher in the high-risk group. Mutation characteristics are considered markers of cancer drug sensitivity [55]. Patients with EGFR mutations are preferentially treated with EGFR inhibitors, followed by drug adjustments based on the development of resistance [56,57]. This strategy led us to investigate the chemotherapeutic sensitivity of patients among risk groups. Interestingly, the IC50 predictions indicated that the high-risk population might indeed benefit from chemotherapy with drugs such as AZD3759, Osimertinib, and Gefitinib. These findings highlighted the significance of developing more effective and personalized treatment strategies for patients, tailored to their specific risk profiles.

In conclusion, we explored the characteristics of dormant tumor cells in LUAD and constructed a risk score model through systematic analysis. Our prognostic model will be helpful in clinical practice, achieving maximum therapeutic benefits for patients with LUAD through effective stratification and personalized management. However, this study has certain limitations. Future research needs to conduct more in-depth experimental studies on these tumor cell subgroups to confirm the findings and the underlying mechanism of O-glycosylation in tumor dormancy.

## 4. Materials and Methods

### 4.1. Acquisition and Processing of Single-Cell Data

The single-cell data utilized in this study were derived from an article published by Philip Bischoff and other researchers titled “Single-cell RNA sequencing reveals distinct tumor microenvironmental patterns in lung adenocarcinoma” [21]. Single-cell analysis was conducted using “Seurat v5.0.3” [58]. In the quality control step, cells with detected gene counts greater than 300, ribosomal protein-encoding gene expression proportions greater than 3%, mitochondrial gene expression proportions less than 20%, and haemoglobin gene expression proportions less than 0.1% were retained. The “NormalizeData” function was employed for standardizing the single-cell dataset, while the “FindVariableFeatures” function identified 3000 variable features within the dataset. The “ScaleData” function was utilized to scale the data, mitigating the effects of varying sequencing depths across different cells. Cell clustering and cell type identification were performed using the “FindClusters” function. Cell type annotation genes were obtained from Cell Marker 2.0 (http://117.50.127.228/CellMarker/, accessed on 1 June 2024). Forty-six tumor dormancy genes, including *GAS6*, *AXL*, *MERTK*, *BHLHE41*, *NR2F1*, *FOXM1*, and *SMAD3*, were identified from published literature. Using the cell cycle genes provided by the “Seurat” package, we calculated the cell cycle scores for each cell and classified them using the “CellCycleScoring” function. Additionally, we employed the “CytoTRACE v0.3.3” function [59] to compute CytoTRACE scores for malignant cells. When the score ranged from 0 to 1, higher scores indicated a stronger stem cell phenotype (less differentiated). Differential gene analysis was conducted using the “FindMarkers” function, and the “AddModuleScore” was used for calculating gene set scores. Tools such as “hdWGCNA”, “cowplot” and “patchwork” were applied for hdWGCNA of the single-cell data. The Monocle2 package was used for single-cell trajectory analysis, followed by the selection of genes with significant expression changes for further analysis via Branch Expression Analysis Modelling (BEAM) [60,61]. The “CellChat v1.5.0” [62] was used for exploring intercellular communication and molecular mechanisms in single-cell data. The “netVisual” series of functions was utilized for visualization purposes. Basically, we used the “identifyOverExpressedGenes” function to find genes that are highly expressed in certain cell types, and then the “identifyOverExpressedInteractions” function helped us figure out which ligand-receptor interactions these genes are involved in. Next, the “computeCommunProb” function calculated how likely it is that cells are communicating with each other, and the “computeCommunProbPathway” function estimated these probabilities for different signaling pathways. Finally, we integrated these communication networks using the “aggregateNet” function and visualized the interactions and pathways with functions from the netVisual series.

### 4.2. Data Collection

Gene expression data, clinical information, and follow-up data for LUAD patients were acquired from the TCGA database through the UCSC Xena platform (https://xenabrowser.net/, accessed on 1 May 2024). After excluding samples with incomplete clinical information, we successfully gathered data for 497 LUAD patients from the TCGA, ensuring the completeness and scientific integrity of the dataset. This comprehensive dataset enables robust analysis and insights into the genetic and clinical landscape of lung adenocarcinoma, facilitating advanced research into its pathogenesis and potential therapeutic targets. This study also utilized normal lung tissue data from the GTEx database (n = 288), which is known for its comprehensive atlas of normal tissue, to compare normal and abnormal or diseased states. We downloaded the transcriptomic and clinical data of two independent cohorts, GSE72094 (398 samples) and GSE31210 (246 samples), from the GEO database to validate our data.

### 4.3. Identification of DEGs

We utilized the “limma” package [63] to identify DEGs among risk groups, and GEO2R (https://www.ncbi.nlm.nih.gov/geo/geo2r/?acc=GSE131594, accessed on 1 June 2024) was used to analyze DEGs in GSE131594. The criteria for determining DEGs were a |log (2) fold change| > 1 and an adjusted *p* < 0.05. This analysis facilitates the understanding of gene expression changes associated with disease treatment efficacy and risk stratification in the context of this study.

### 4.4. GO and KEGG Analysis

In this study, the “clusterProfiler v4.10.0”, “org.Hs.eg.db v3.18.0”, “enrichplot v1.22.0”, and “ggplot2 v3.5.1” et al. packages were used to analyze the functions of the dormancy-related DEGs or the DEGs between the high-risk and low-risk groups. Furthermore, an adjusted *p* < 0.05 was used to filter the functional candidates.

### 4.5. Gene Set Variation Analysis

GSVA is a robust method for analyzing gene sets and identifying enriched biological pathways [64]. In our study, to perform enrichment analysis, we downloaded the “GOBP_PROTEIN_O_LINKED_GLYCOSYLATION” gene set (n  =  99) from MSigDB v7.5 (https://www.gsea-msigdb.org/gsea/msigdb/human/search.jsp, accessed on 1 June 2024). We then utilized the GSCA website (https://guolab.wchscu.cn/GSCA, accessed on 1 June 2024) to calculate gene set scores. To ensure the statistical significance of our results, we set the threshold for significant enrichment with an adjusted P value of less than 0.05.

### 4.6. Construction of the Prognostic Model and Validation

To construct a prognostic model related to dormancy in TCGA-LUAD samples, we utilized GSVA and machine learning to evaluate the samples. We used the “cv.glmnet” function from the “glmnet” package to perform LASSO regression and identify the best regularization parameter, λ. To ensure consistent results, we set the random seed to 3. The model was built using the “cv.glmnet” function with family = “cox” to fit a Cox proportional hazards model for survival analysis. We then conducted a 10-fold cross-validation to select the optimal λ value (cvfit$lambda.min) and adjusted the model’s regularization strength accordingly. The C-index was calculated using the “survConcordance” function from the “survival” package to evaluate the accuracy and consistency of the model. These C-index values helped us assess the model’s performance. Ultimately, we narrowed down 53 ORDGs to 18, creating a new prognostic gene signature. The risk score for each patient was calculated using the following formula:

Risk score = ∑ (Expi × Coef)

where Expi represents the expression level of each marker gene, and Coef represents the corresponding coefficient. The predictive performance of this model was assessed using an ROC curve, risk plots, and the C-index. The reliability of the prognostic features was validated using an external validation cohort (GSE72094). Additionally, we used the “survival”, “rms”, and “regplot” et al. packages to develop a nomogram for predicting the OS rates. The signature was calibrated to evaluate its consistency with actual outcomes, providing a comprehensive tool for assessing patient prognosis in lung adenocarcinoma patients based on gene expression related to dormancy processes.

### 4.7. Tumor Microenvironment

Using expression data, we evaluated the tumor immune microenvironment and immunogenomic characteristics of LUAD samples. The ESTIMATE algorithm provides an assessment of the tumor environment, estimates tumor purity, and quantifies the abundance of infiltrating stromal and immune cells. Furthermore, we applied the CIBERSORT algorithm, a deconvolution method based on linear support vector regression, which uses LUAD gene expression profiles to quantify the composition of 22 types of TIICs.

### 4.8. Mutation Analysis

Somatic mutation data for 497 LUAD patients were analyzed to determine the TMB, a potential biomarker for predicting responses to immunotherapy. The mutation data were downloaded from the cBioPortal database (https://www.cbioportal.org/, accessed on 1 June 2024). Using the “maftools v2.18.0” package, waterfall plots were generated to depict the mutation landscape in the high-risk and low-risk groups of LUAD patients.

### 4.9. Prediction of Drug Sensitivity

The 50% inhibitory concentration (IC50) for the different risk groups was calculated using the “oncoPredict v1.2” package, and ridge regression was used to predict their chemotherapy outcomes [65]. The GDSC2 dataset, comprising gene expression profiles and drug sensitivity data for numerous human cancer lines, was employed to investigate the drug responsiveness.

### 4.10. Cell Culture

The lung cancer cell lines (A549 and H1299) and the normal pulmonary epithelial cell line (Beas-2b) were purchased from the American Type Culture Collection (ATCC). The specific conditions of cell culture referred to our previous studies [66]. To establish tumor dormancy [67,68], A549 or H1299 cells were cocultured in reduced growth factor BME with HUVECs in DMEM supplemented with 2% FBS, 100 U/mL of penicillin, 100 μg/mL of streptomycin, 1 g/L of glucose, 4 mM of L-Glutamine, 1 mM of sodium pyruvate, and 1,500 mg/L of sodium bicarbonate.

### 4.11. RNA Extraction and Quantitative Real-Time PCR (qRT-PCR)

The specific experiments were performed as previously described [66]. The qRT-PCR primers are listed in Table 1.

### 4.12. Cell Viability Assay

Predetermined time after cells seeded, 50 μl of CCK 8 (Dojindo, Kumamoto, Japan) reagent was added to 450 μL of mixture and cultured at 37 °C for 2 h. The absorbance of each sample was assessed at 450 nm using an automated plate reader (Thermo Fisher, Waltham, MA, USA).

### 4.13. Statistical Analysis

Statistical analyses were conducted using R (version 4.3.2) and GraphPad Prism 8 software. The Wilcoxon rank test was utilized to analyze the distribution and expression of risk scores, stromal scores, immune scores, tumor purity, drug sensitivity, and TMB across different groups. Spearman’s method was used to evaluate correlations. *p* < 0.05 was considered to indicate statistical significance. * *p* < 0.05, ** *p* < 0.01, *** *p* < 0.001, **** *p* < 0.0001, ns: not significant.

## Figures and Tables

**Table 1:** The primer sequences for qRT-PCR.

Gene	Forward Primer (5′-3′)	Reverse Primer (5′-3′)
*PTTG1IP*	GTCTGGACTACCCAGTTACAAGC	CGCCTCAAAGTTCACCCAA
*EFNB2*	TATGCAGAACTGCGATTTCCAA	TGGGTATAGTACCAGTCCTTGTC
*TNFRSF11A*	AGATCGCTCCTCCATGTACCA	GCCTTGCCTGTATCACAAACTTT
*GAPDH*	GAACATCATCCCTGCCTCTACT	CCTGCTTCACCACCTTCTTG

## Data Availability

All data are available in a public, open access repository. The raw LUAD scRNA-seq data were obtained from https://codeocean.com/capsule/8321305/tree/v1, accessed on 1 May 2024. The data analyzed in the current study, GSE131907 (https://www.ncbi.nlm.nih.gov/geo/query/acc.cgi?acc=GSE131594, accessed on 1 May 2024), GSE72094 (https://www.ncbi.nlm.nih.gov/geo/query/acc.cgi?%20And%20acc%20=%20GSE72094, accessed on 1 May 2024), and GSE31210 (https://www.ncbi.nlm.nih.gov/geo/query/acc.cgi?acc=GSE31210, accessed on 1 May 2024), are available from the GEO database. R and other custom scripts for analyzing the data are available upon reasonable request.

## Supplementary Materials

The following supporting information can be downloaded at: https://www.mdpi.com/article/10.3390/ijms25179502/s1.
