# Supplementary material for: Deciphering Dormant Cells of Lung Adenocarcinoma: Prognostic Insights from O-glycosylation-Related Tumor Dormancy Genes Using Machine Learning
Source: Int J Mol Sci. 2024 Aug 31;25(17):9502. doi: 10.3390/ijms25179502 (PMC11395112; doi:10.3390/ijms25179502)
Supplement: Supplementary file 1 [file ijms-25-09502-s001.zip › Supplementary materials.pdf]

Supplementary figures:

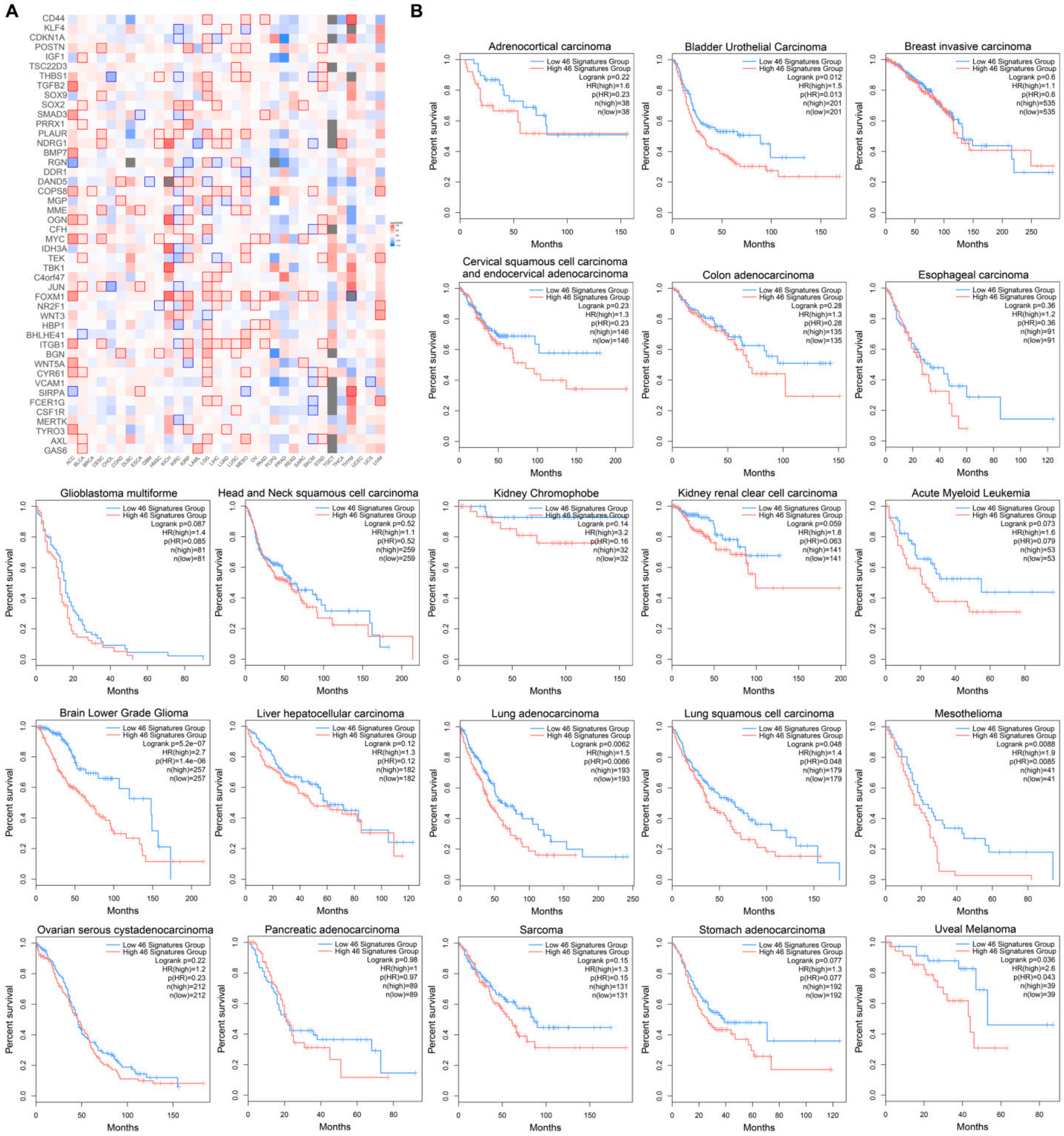

Figure S1. Pan-cancer analysis of tumor dormancy signature. (A) Hazard ratio analysis of the tumor dormancy-related genes in different tumors. (B) K-M curves of the tumor dormancy signature in different tumors.

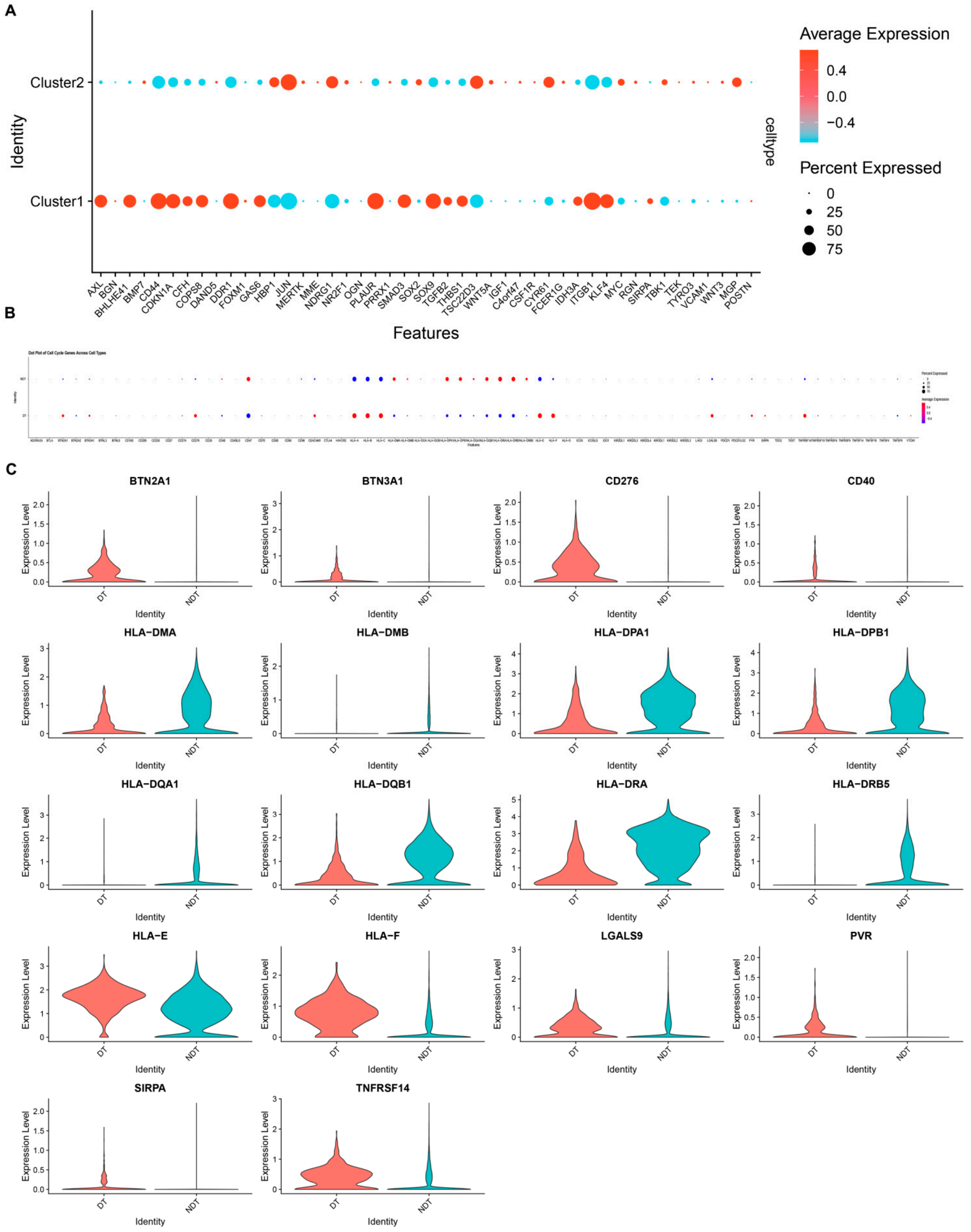

Figure S2. Immune checkpoint analysis of the DT and NDT clusters. (A) The bubble plots of the expression of dormancy-related genes in two cells clusters. (B) Differences in expression levels of 67 immune checkpoints. (C) The violin plots of immune checkpoints genes in DT and NDT cluster.

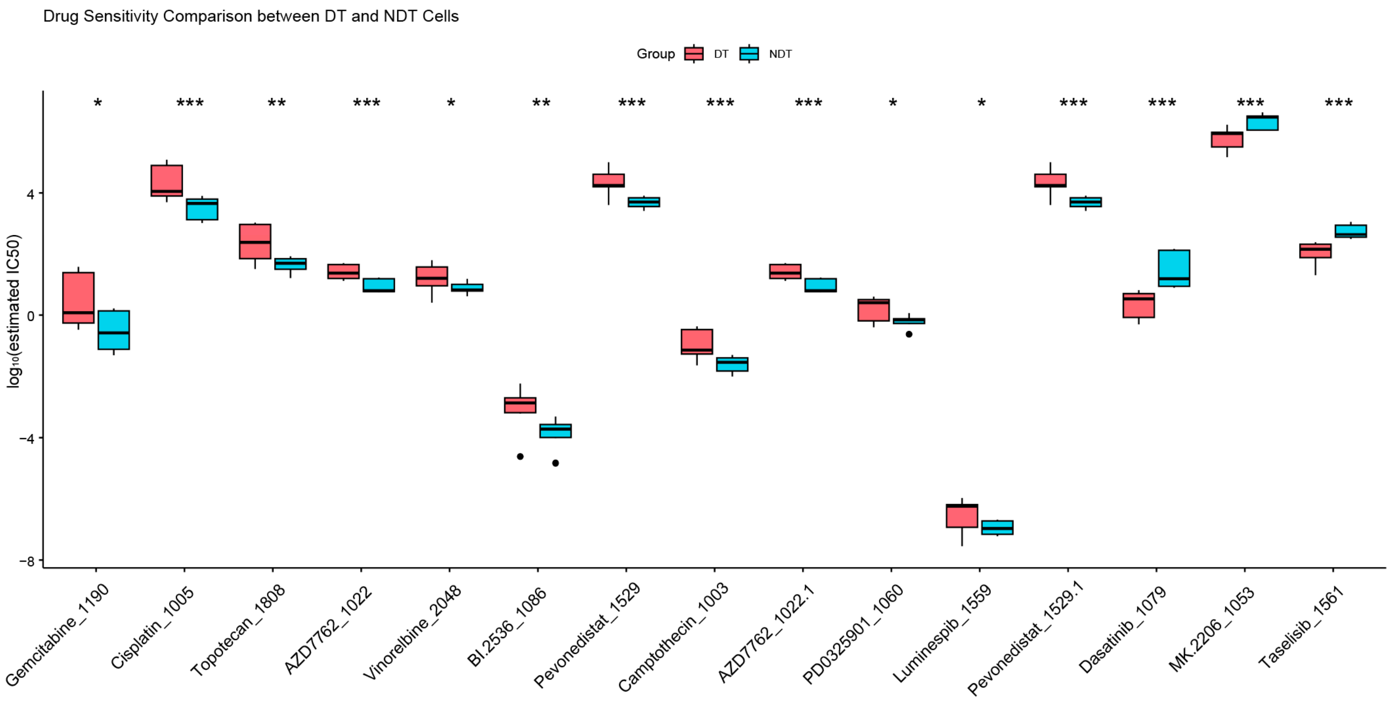

Figure S3. Drug sensitivity prediction between DT and NDT groups in GSE131594.

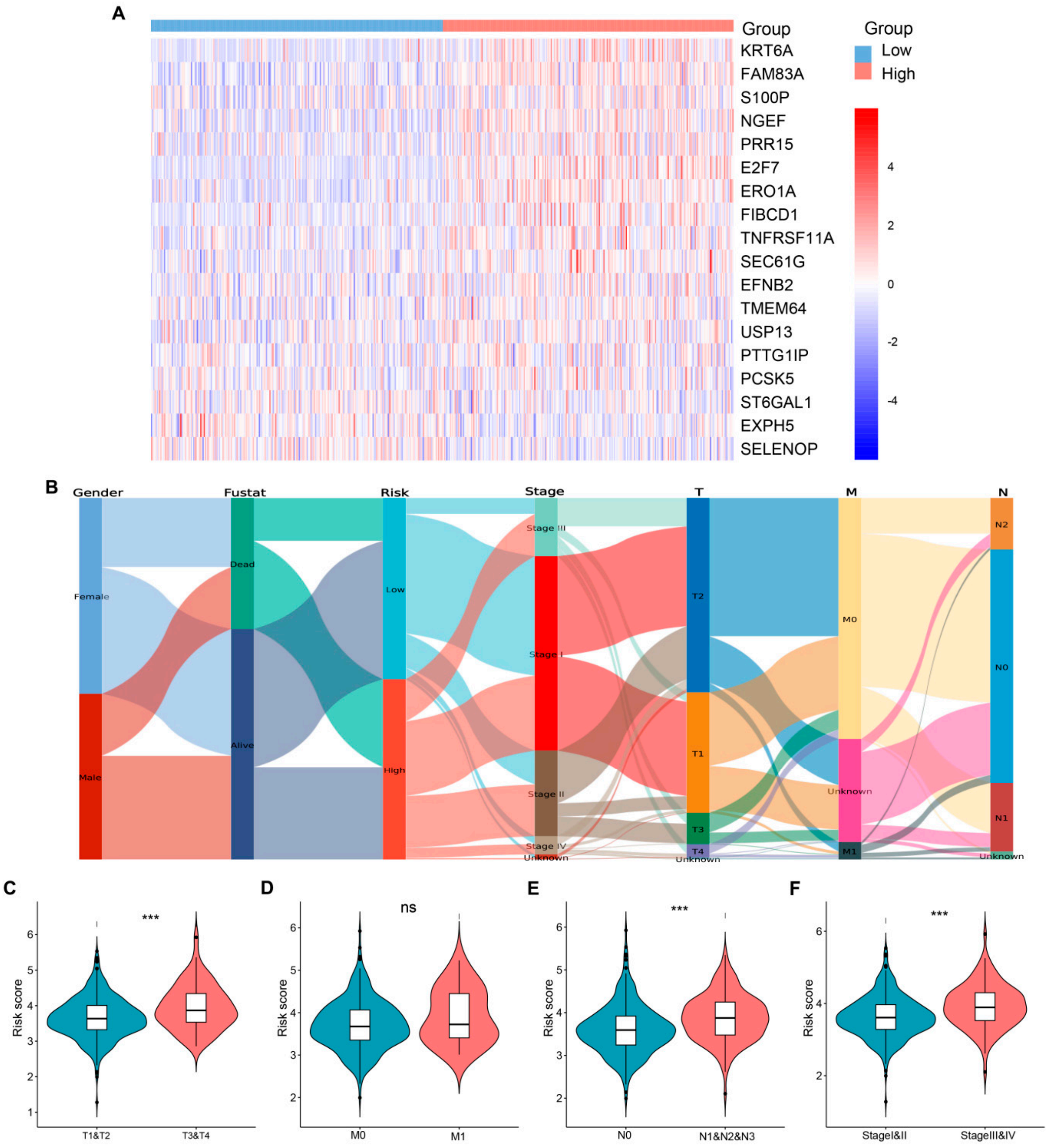

Figure S4. The relationship between risk score and the clinical characteristics in LUAD. (A) The heatmaps of the distribution of 18 model genes in the two groups. (B) Sankey diagram showed the correspondence of gender, survival status, risk score, stage diagnoses, pathologic T, pathologic M and pathologic N. (C) Comparison of risk scores for patients with different pathologic (T1&T2 and T3&T4) tumors. (D) Comparison of risk scores for patients with different pathologic (M0 and M1) tumors. (E) Comparison of risk scores for patients with different pathologic (N0 and N1&N2&N3) tumors. (F) Comparison of risk scores for patients with different stages (Stage I&II and Stage III&IV) tumors.
